# Evaluation of Continuous Tumor-Size–Based End Points as Surrogates for Overall Survival in Randomized Clinical Trials in Metastatic Colorectal Cancer

**DOI:** 10.1001/jamanetworkopen.2019.11750

**Published:** 2019-09-20

**Authors:** Tomasz Burzykowski, Elisabeth Coart, Everardo D. Saad, Qian Shi, Dirkje W. Sommeijer, Carsten Bokemeyer, Eduardo Díaz-Rubio, Jean-Yves Douillard, Alfredo Falcone, Charles S. Fuchs, Richard M. Goldberg, J. Randolph Hecht, Paulo M. Hoff, Herbert Hurwitz, Fairooz F. Kabbinavar, Miriam Koopman, Timothy S. Maughan, Cornelis J. A. Punt, Leonard Saltz, Hans-Joachim Schmoll, Matthew T. Seymour, Niall C. Tebbutt, Christophe Tournigand, Eric Van Cutsem, Aimery de Gramont, John R. Zalcberg, Marc Buyse

**Affiliations:** 1 International Drug Development Institute, Louvain-la-Neuve, Belgium; 2 Hasselt University, Diepenbeek, Belgium; 3 Division of Biomedical Statistics and Informatics, Mayo Clinic, Rochester, Minnesota; 4 The University of Sydney, Camperdown, New South Wales, Australia; 5 Academic Medical Centre, Amsterdam, the Netherlands; 6 Flevohospital, Almere, the Netherlands; 7 Department of Internal Medicine II and Clinic, University of Hamburg, Hamburg, Germany; 8 Hospital Clinico San Carlos and Centro de Investigación Biomédica en Red Cáncer, CIBERONC, Madrid, Spain; 9 Centre René Gauducheau, St Herblain, France; 10 University Hospital S Chiara, Pisa, Italy; 11 Dana-Farber Cancer Institute, Boston, Massachusetts; 12 West Virginia University Cancer Institute, Morgantown; 13 David Geffen School of Medicine, University of California, Los Angeles; 14 Instituto de Câncer do Estado de São Paulo, São Paulo, Brazil; 15 Genentech, South San Francisco, California; 16 Department of Medical Oncology, University Medical Centre Utrecht, Utrecht University, Utrecht, the Netherlands; 17 Cancer Research UK and the Medical Research Council Oxford Institute for Radiation Oncology, Oxford, United Kingdom; 18 Amsterdam University Medical Centrum, Department of Medical Oncology, University of Amsterdam, Amsterdam, the Netherlands; 19 Memorial Sloan-Kettering Cancer Center, New York, New York; 20 Martin-Luther University, Halle, Germany; 21 St James's Hospital, University of Leeds, Leeds, United Kingdom; 22 Austin Health, Heidelberg, Victoria, Australia; 23 Hôpital Henri Mondor, Creteil, France; 24 Division of Digestive Oncology, University Hospitals Gasthuisberg Leuven, Leuven, Belgium; 25 Katholieke Universiteit, Leuven, Belgium; 26 Franco-British Institute, Levallois-Perret, France; 27 School of Public Health and Preventative Medicine, Monash University, Melbourne, Australia; 28 International Drug Development Institute Inc, San Francisco, California

## Abstract

**Importance:**

Tumor measurements can be used to estimate time to nadir and depth of nadir as potential surrogates for overall survival (OS).

**Objective:**

To assess time to nadir and depth of nadir as surrogates for OS in metastatic colorectal cancer.

**Design, Setting, and Participants:**

Pooled analysis of 20 randomized clinical trials within the Aide et Recherche en Cancerologie Digestive database, which contains academic and industry-sponsored trials, was conducted. Three sets of comparisons were performed: chemotherapy alone, antiangiogenic agents, and anti–epidermal growth factor receptor agents in first-line treatment for patients with metastatic colorectal cancer.

**Main Outcomes and Measures:**

Surrogacy of time to nadir and depth of nadir was assessed at the trial level based on joint modeling of relative tumor-size change vs baseline and OS. Treatment effects on time to nadir and on depth of nadir were defined in terms of between-arm differences in time to nadir and in depth of nadir, and both were assessed in linear regressions for their correlation with treatment effects (hazard ratios) on OS within each set. The strengths of association were quantified using sample-size–weighted coefficients of determination (*R*^2^), with values closer to 1.00 indicating stronger association. At the patient level, the correlation was assessed between modeled relative tumor-size change and OS.

**Results:**

For 14 chemotherapy comparisons in 4289 patients, the *R*^2^ value was 0.63 (95% CI, 0.30-0.96) for the association between treatment effects on time to nadir and OS and 0.08 (95% CI, 0-0.37) for depth of nadir and OS. For 11 antiangiogenic agent comparisons (4854 patients), corresponding values of *R*^2^ were 0.25 (95% CI, 0-0.72) and 0.06 (95% CI, 0-0.35). For 8 anti–epidermal growth factor receptor comparisons (2684 patients), corresponding values of *R*^2^ were 0.24 (95% CI, 0-0.83) and 0.21 (95% CI, 0-0.78).

**Conclusions and Relevance:**

In contrast with early reports favoring depth of response as a surrogate, these results suggest that neither time to nadir nor depth of nadir is an acceptable surrogate for OS in the first-line treatment of metastatic colorectal cancer.

## Key Points

**Question:**

Can end points based on the kinetics of tumor size after treatment be used as surrogates for overall survival in metastatic colorectal cancer?

**Findings:**

In this pooled analysis of data from 20 randomized clinical trials, time to nadir and depth of nadir were modeled and assessed as potential surrogates for overall survival at the patient and trial levels. The associations found were weak or moderate; there were notable differences in tumor-size kinetics between antiangiogenic agents and anti–epidermal growth factor receptor agents.

**Meaning:**

The implications of these results for early drug development and clinical practice are unclear and warrant further studies; the findings of this study reinforce the need to develop more reliable end points that reflect tumor biology and patient benefit.

## Teaser

This study examines the use of time to nadir and depth of nadir as surrogate end points for overall survival in phase 3 randomized clinical trials evaluating first-line treatment in patients with metastatic colorectal cancer.

## Introduction

The availability of active treatments for use in subsequent lines have called into question the use of overall survival (OS) as a primary end point in phase 3 trials on first-line therapy for metastatic colorectal cancer (mCRC).^1^ As a result, there has been a long-standing interest in developing and validating surrogate end points for OS in this setting.^2,3^ Such validation requires demonstration of a strong association between the surrogate and the final end point at the patient level (ie, patients with improvements in the surrogate end point also tend to have improvements in the final end point) and a strong association between the treatment effects on the surrogate end point and the final end point (the trial-level association).^4^ Tumor-size–based end points have generated interest in the search for early treatment end points in mCRC.^5,6,7,8,9^ These end points may be categorical or continuous and, among the latter type, the end point receiving the most attention has been the depth of response, defined as the maximum percent tumor shrinkage during treatment. In work published in abstract form, the depth of response was found to be associated with OS at the patient level in first-line cetuximab-based therapy.^10^ That study was based on 2 randomized trials and did not assess the trial-level surrogacy. To obtain a more in-depth view of this question, we assessed the individual- and trial-level surrogacy for OS of 2 continuous tumor-size–based end points in first-line treatment of mCRC.

## Methods

### Trial Selection and Definition of Contrasts

Tumor measurements and OS data were available from 20 first-line randomized clinical trials in mCRC within the Aide et Recherche en Cancerologie Digestive (ARCAD) database (Table 1).^11,12,13,14,15,16,17,18,19,20,21,22,23,24,25,26,27,28,29,30^ To evaluate the trial-level surrogacy, unbiased estimates of treatment effects are needed; hence, the clinical trial database was used. While our analysis used data from several randomized clinical trials, it is not a classic meta-analysis attempting to evaluate pooled estimates of treatment effects. As such, the study follows the recently published Reporting of Surrogate Endpoint Evaluation Using Meta-analyses (ReSEEM) Reporting Guidelines^31^ rather than the Preferred Reporting Items for Systematic Reviews and Meta-analyses (PRISMA) guidelines.

**Table 1.** Control and Experimental Arms for the 3 Treatment Classes Included in the Analysis

Study	Contrast	Treatment (Sample Size, No.)^a^^,^^b^	
		Control	Experimental
**Chemotherapy Alone (n = 4289)**			
Díaz-Rubio et al,^11^ 2007	03-TTD-01	FUOX (136)	XELOX (137)
Fuchs et al,^12^ 2007	BICC-C A	FOLFIRI (28)	Modified IFL (61)
Fuchs et al,^12^ 2007	BICC-C B	FOLFIRI (27)	CAPIRI (54)
Tournigand et al,^13^ 2004	C97-3	FOLFIRI → FOLFOX6 (79)	FOLFOX6 → FOLFIRI (86)
Koopman et al,^14^ 2007	CAIRO1	Capecitabine → irinotecan → XELOX (295)	CAPIRI → XELOX (291)
Seymour et al,^15^ 2007	FOCUS A	Fluorouracil/leucovorin → irinotecan (231)	FOLFIRI (231)
Seymour et al,^15^ 2007	FOCUS B	Fluorouracil/leucovorin → I (227)	FOLFOX (235)
Seymour et al,^16^ 2011	FOCUS2 A	Fluorouracil/leucovorin (74)	FOLFOX (80)
Seymour et al,^16^ 2011	FOCUS2 B	Capecitabine (77)	XELOX (78)
Falcone et al,^17^ 2007	GONO	FOLFIRI (33)	FOLFOXIRI (46)
Saltz et al,^18^ 2008	N016966 A	FOLFOX4 (284)	XELOX (284)
Saltz et al,^18^ 2008	N016966 B	FOLFOX4 (160)	XELOX (162)
Goldberg et al,^19^ 2004	N9741 A	IFL (149)	FOLFOX (300)
Goldberg et al,^19^ 2004	N9741 B	rIFL (171)	Irinotecan, oxaliplatin (273)
**Antiangiogenic Agents (n = 4854)**			
Tebbutt et al,^20^ 2010	AGITG (MAX) A	Capecitabine (75)	Capecitabine + bevacizumab (140)
Tebbutt et al,^20^ 2010	AGITG (MAX) B	Capecitabine (68)	Capecitabine + bevacizumab + mitomycin C (138)
Hurwitz et al,^21^ 2004	AVF2107g A	IFL (187)	IFL + bevacizumab (363)
Hurwitz et al,^21^ 2004	AVF2107g B	IFL (176)	Fluorouracil/leucovorin + bevacizumab (98)
Kabbinavar et al,^22^ 2005	AVF2192g	Fluorouracil/leucovorin (80)	Fluorouracil/leucovorin + bevacizumab (95)
Hoff et al,^23^ 2012	HORIZON II A	FOLFOX/XELOX (171)	FOLFOX/XELOX + cediranib (474)
Hoff et al,^23^ 2012	HORIZON II B	FOLFOX/XELOX (170)	FOLFOX/XELOX + cediranib (198)
Schmoll et al,^24^ 2012	HORIZON III A	FOLFOX + cediranib (654)	FOLFOX + bevacizumab (329)
Schmoll et al,^24^ 2012	HORIZON III B	FOLFOX + cediranib (172)	FOLFOX + bevacizumab (330)
Saltz et al,^18^ 2008	N016966 C	FOLFOX4 (161)	FOLFOX4 + bevacizumab (310)
Saltz et al,^18^ 2008	N016966 D	XELOX (156)	XELOX + bevacizumab (309)
**Anti-EGFR Agents (n = 2684)**			
Tol et al,^25^ 2009	CAIRO2	CAPOX + bevacizumab (126)	CAPOX + bevacizumab + cetuximab (128)
Maughan et al,^26^ 2011	COIN A	Fluorouracil/leucovorin/oxaliplatin (99)	Fluorouracil/leucovorin/oxaliplatin + cetuximab (82)
Maughan et al,^26^ 2011	COIN B	Capecitabine/oxaliplatin (189)	Capecitabine/oxaliplatin + cetuximab (184)
Van Cutsem et al,^27^ 2009	CRYSTAL	FOLFIRI (324)	FOLFIRI + cetuximab (291)
Bokemeyer et al,^28^ 2009	OPUS	FOLFOX (88)	FOLFOX + cetuximab (76)
Hecht et al,^29^ 2009	PACCE (C249) A	Oxaliplatin based + bevacizumab (188)	Oxaliplatin based + bevacizumab + panitumumab (178)
Hecht et al,^29^ 2009	PACCE (C249) B	Irinotecan based + bevacizumab (51)	Irinotecan based + bevacizumab + panitumumab (50)
Douillard et al,^30^ 2010	PRIME (C203)	FOLFOX4 (318)	FOLFOX4 + panitumumab (312)

Abbreviations: AGITG, Australasian Gastro-Intestinal Cancer Trials Group; anti-EGFR, anti–epidermal growth factor receptor; BICC, Bolus, Infusional, or Capecitabine With Camptosar-Celecoxib; CAPIRI, capecitabine, irinotecan; CAPOX, capecitabine, oxaliplatin; FOCUS, Fluoxetine or Control Under Supervision; FOLFIRI, fluorouracil, leucovorin, irinotecan; FOLFOX, fluorouracil, leucovorin, oxaliplatin; FOLFOXIRI, fluorouracil, leucovorin, oxaliplatin, irinotecan; FUOX, fluorouracil, oxaliplatin; GONO, Gruppo Oncologico Nord Ovest; IFL, irinotecan, fluorouracil, leucovorin; MAX, Mitomycin C, Avastin and Xeloda; rIFL, reduced-dose irinotecan, fluorouracil, leucovorin; PACCE, Panitumumab Advanced Colorectal Cancer Evaluation; PRIME, Panitumumab Randomized Trial in Combination With Chemotherapy for Metastatic Colorectal Cancer to Determine Efficacy; TTD, Spanish Cooperative Group for Gastrointestinal Tumor Therapy; XELOX, capecitabine, oxaliplatin; and →, subsequently.

^a^ Sample sizes may differ from those reported in the original publications owing to exclusion of patients in the present analysis (see Methods section for details).

^b^ Numbers with the combination regimens (eg, FOLFOX6) are used by the original developers of these regimens to denote subsequent versions and improvements in the administration schedule.

Tumor measurements consisted of the longest diameters of target lesions, used in the original trials according to the Response Evaluation Criteria in Solid Tumors (RECIST) guideline, version 1.1.^32^ Eight trials involved only chemotherapy; of the 12 trials that had at least 1 biological agent, 6 evaluated antiangiogenic (anti-ANG) agents as the only biological, 4 investigated an anti–epidermal growth factor receptor (anti-EGFR) agent as the only biological, and 2 trials had both an anti-ANG and anti-EGFR agent. The analysis was based on comparisons between 2 arms (henceforth termed *contrasts*) nested within trials, with control and experimental arms defined according to historical evolution. An exception to this rule was made for HORIZON III,^24^ for which the cediranib arm was considered as control to have bevacizumab as the uniform experimental intervention for anti-ANG agents (Table 1). For 8 trials with more than 2 arms, each experimental arm was compared with a control arm created by randomly splitting the set of patients originally randomized to the control arm. This procedure was applied to avoid including each patient twice in the analysis, which would artificially induce a correlation that would confound the associations under investigation.

### Statistical Analysis

Target lesions measured up to 24 months after randomization were used, as 98% of the available postbaseline measurements were made within 24 months. Individual trials had tumor-assessment schedules that varied between 6 and 12 weeks, but this variation does not influence the models used here. Overall survival was defined as the time from randomization to death from any cause, with censoring of data from patients who were alive at the last contact date. Separate analyses were conducted for chemotherapy-only contrasts, anti-ANG-agent contrasts, and anti-EGFR-agent contrasts. Because *KRAS* (OMIM *190070) is a predictive biomarker for anti-EGFR treatment, only patients with wild-type *KRAS* were considered in contrasts evaluating the effects of such treatments. For trials of different treatment sequences, only contrasts for which the 2 arms testing different regimens at the beginning of the treatment sequence were analyzed. For the Bolus, Infusional, or Capecitabine With Camptosar-Celecoxib trial,^12^ treatment arms with celecoxib were not analyzed.

Tumor-size measurements (the sum of all target lesions) were modeled using the relative tumor-size change (RTSC) vs baseline, defined (for time *t*) as follows: RTSC(*t*) = (tumor size at time *t* – tumor size at baseline) / (tumor size at baseline).

**Figure 1.**
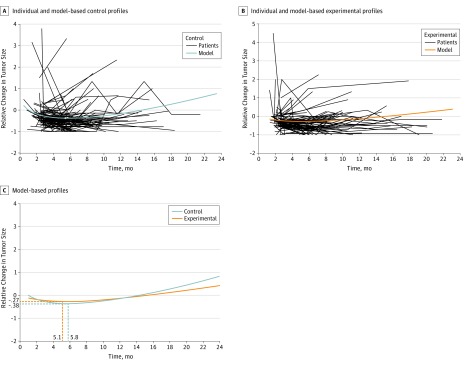
Longitudinal Profiles A, Relative tumor-size changes over time for individual patients and the model-based estimated profile for the control group. B, Relative tumor-size changes over time for individual patients and the model-based estimated profile for the experimental group. C, Based on the model-based profiles, the nadir for the control arm is estimated to occur at 5.8 months, with the depth of nadir −0.38 (ie, a 38% reduction of the tumor mass relative to baseline). Corresponding figures for the experimental arm are 5.1 months for the time of occurrence of the nadir and −0.27 (ie, 27% reduction of the tumor mass relative to baseline) for the depth of nadir. Consequently, the effect of experimental treatment in terms of time to nadir and depth of nadir is equal to 5.1 − 5.8 = -0.7 months and −0.27 − (−0.38) = 0.11. That is, in the experimental arm, the nadir occurs earlier and is 11% smaller (ie, less deep) than in the control arm.

Repeated values of RTSC and the time to death were analyzed in joint models.^33,34^ In particular, RTSC measures were analyzed by linear mixed-effects models with contrast-specific fixed and random linear and square-root time effects. Overall survival was analyzed by proportional hazards models that included the random effects from the RTSC model to account for the association between RTSC and survival time. Based on the joint models, treatment effects on RTSC and OS were estimated. For OS, the effects were estimated using the natural logarithm of the hazard ratio (HR) obtained from the proportional hazards model (logHR). For RTSC, the outcomes were defined based on the mean treatment-specific time profiles estimated using the linear mixed-effects model. In particular, for each profile, the nadir (ie, the local minimum RTSC value) was obtained, together with the time at which the nadir took place. Treatment effects were then defined in terms of differences in time to nadir and differences in depth of nadir; the latter variable is analogous to depth of response but is estimated from the model rather than coming directly from patient data. Figure 1 illustrates the calculation of longitudinal profiles for 1 of the contrasts. For differences in time to nadir, negative values indicate that the nadir occurs earlier with experimental treatment; for differences in depth of nadir, negative values indicate that the nadir is deeper with experimental treatment.

To assess the validity of time to nadir and depth of nadir as surrogates for OS, we applied the correlation approach.^33^ Specifically, a linear regression was fitted to the estimated pairs of treatment effects on time to nadir or depth of nadir and OS. The regression was weighted by the contrast-specific sample size. The coefficient of determination (*R*^2^) was used to quantify the strength of association at the trial level between the treatment effects on time to nadir or depth of nadir and OS. An *R*^2^ value greater than 0.75 was considered an indicator of good surrogacy.^35,36^ We also quantified the strength of association at the individual level between RTSC and OS. With this aim, we measured the correlation between the individual random effects included in the linear mixed-effects model for RTSC and the proportional hazards model for OS using a correlation coefficient, denoted by *R*(*t*).^33^ This correlation coefficient is a time-dependent measure, since the association between RTSC and the death process can be defined relative to any time over the course of tumor-size measurements. In the analysis, 2-sided 95% CIs were used. Analyses were conducted with SAS, version 9.4 (SAS Institute Inc) and Stata, version 13.1 (StataCorp LLC).

## Results

### Chemotherapy Alone

There were 6224 patients in the ARCAD database enrolled in 9 trials eligible for this analysis (8 trials involving only chemotherapy and 1 trial that included bevacizumab but provided chemotherapy-alone contrasts). After excluding patients without any tumor-size information or with tumor-size measurements available only more than 24 months after randomization, 4289 patients (68.9%) could be analyzed (Table 1). Such patients were grouped in 14 contrasts, with the median follow-up per trial ranging from 14 to 128 months. eFigure 1A in the Supplement presents the Kaplan-Meier OS curves for these 14 contrasts, with the corresponding HRs presented in Table 2.

**Table 2.** Estimated Time to Nadir and Depth of Nadir

Contrast	Time to Nadir, mo^a^			Depth of Nadir, m^b^			HR for OS^d^
	Control	Experimental	Treatment Effect^c^	Control	Experimental	Treatment Effect^c^	
**Chemotherapy Alone**							
03-TTD-01	5.81	5.09	−0.72	−0.38	−0.27	0.11	1.06
BICC-C A	5.89	5.26	−0.64	−0.32	−0.36	−0.04	1.07
BICC-C C	11.04	6.52	−4.52	−0.46	−0.29	0.17	1.57
C97-3	5.32	4.82	−0.50	−0.40	−0.34	0.06	0.83
CAIRO1	2.72	3.73	1.01	−0.06	−0.24	−0.18	0.80
FOCUS A	0.66	3.05	2.39	−0.12	−0.09	0.02	0.88
FOCUS B	2.97	2.70	−0.27	0.23	−0.26	−0.49	0.93
FOCUS2 A	1.17	2.00	0.83	0.40	−0.07	−0.47	1.01
FOCUS2 B	0.05	NA	NA	− 0.01	NA	NA	0.99
GONO	6.37	11.14	4.77	−0.43	−0.66	−0.23	0.78
N016966 A	5.25	4.82	−0.43	−0.38	−0.40	−0.02	0.89
N016966 B	6.17	4.59	−1.58	−0.43	−0.36	0.07	1.16
N9741 A	4.66	7.31	2.65	−0.28	−0.44	−0.16	0.68
N9741 B	4.57	4.75	0.18	−0.24	−0.27	−0.01	0.90
**Antiangiogenic Agents**							
AGITG (MAX) A	3.42	4.13	0.70	−0.15	−0.26	−0.11	0.88
AGITG (MAX) B	3.05	4.82	1.78	−0.11	−0.28	−0.17	1.07
AVF2107g A	4.02	6.34	2.32	−0.26	−0.37	−0.11	0.73
AVF2107g B	3.66	6.66	2.99	−0.21	−0.27	−0.06	0.80
AVF2192g	3.88	4.51	0.63	−0.24	−0.26	−0.02	0.91
HORIZON II A	4.98	5.28	0.30	−0.38	−0.39	−0.01	0.88
HORIZON II B	4.56	5.93	1.37	−0.32	−0.41	−0.09	0.96
HORIZON III A	5.70	6.64	0.93	−0.35	−0.35	0.00	1.09
HORIZON III B	5.38	5.92	0.54	−0.30	−0.34	−0.04	1.00
N016966 C	5.37	6.79	1.42	−0.37	−0.36	0.01	0.85
N016966 D	4.93	6.04	1.10	−0.33	−0.36	−0.03	0.82
**Anti-EGFR Agents**							
CAIRO2	6.78	5.21	−1.57	−0.26	−0.33	−0.07	1.13
COIN A	6.37	8.34	1.97	−0.31	−0.40	−0.09	0.76
COIN B	5.82	2.97	−2.85	−0.03	−0.30	−0.27	1.09
CRYSTAL	6.28	8.26	1.98	−0.31	−0.46	−0.16	0.74
OPUS	7.83	10.23	2.40	−0.34	−0.55	−0.22	0.86
PACCE (C249) A	7.40	7.77	0.37	−0.37	−0.31	0.06	1.48
PACCE (C249) B	171.1	7.99	−163.1	−0.78	−0.37	0.41	1.76
PRIME (C203)	8.36	9.22	0.86	−0.40	−0.48	−0.08	0.81

Abbreviations: AGITG, Australasian Gastro-Intestinal Cancer Trials Group; anti-EGFR, anti–epidermal growth factor receptor; BICC, Bolus, Infusional, or Capecitabine With Camptosar-Celecoxib; FOCUS, Fluoxetine or Control Under Supervision; GONO, Gruppo Oncologico Nord Ovest; HR, hazard ratio; OS, overall survival; PACCE, Panitumumab Advanced Colorectal Cancer Evaluation; PRIME, Panitumumab Randomized Trial in Combination With Chemotherapy for Metastatic Colorectal Cancer to Determine Efficacy; TTD, Spanish Cooperative Group for Gastrointestinal Tumor Therapy.

^a^ For differences in time to nadir, negative values indicate that the nadir occured earlier with experimental treatment.

^b^ For differences in depth of nadir, negative values indicate that the nadir was deeper with experimental treatment.

^c^Experimental minus control.

^d^Hazard ratios may differ from those reported in the original publications owing to exclusion of patients in the present analysis and the use of a different modeling framework (a joint model for relative tumor-size change and OS).

eFigure 2A in the Supplement presents the estimated, model-based longitudinal profiles for each contrast in these trials. The corresponding estimates of treatment effects in terms of the differences in time to nadir and depth of nadir are presented in Table 2. There was large variability in the treatment effects, reflecting relatively small and inconsistent differences in the longitudinal profiles (eFigure 2A in the Supplement). For instance, for time to nadir, the estimated treatment effects varied (Table 2) from −4.53 months (BICC-C C) to 4.77 months (Gruppo Oncologico Nord Ovest). For depth of nadir, the range was from −0.49 (FOCUS B) to 0.17 (BICC-C C). For 1 comparison (FOCUS2 B), the effects could not be obtained because the estimated RTSC profile for the experimental arm did not reach a local minimum (the profile was a strictly increasing function of time).

**Figure 2.**
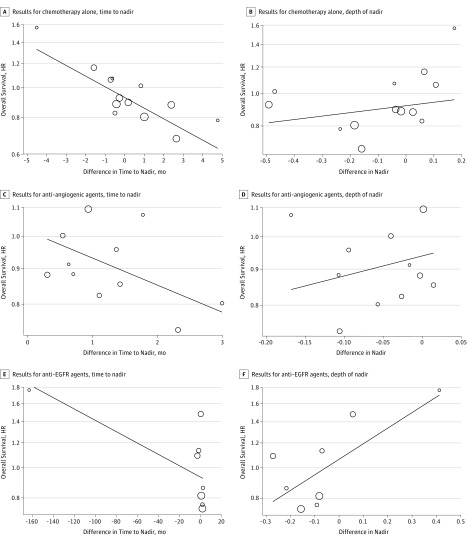
Trial-Level Associations Between Treatment Effects Hazard ratios (HRs) of overall survival associated with time to nadir and depth of nadir in the chemotherapy-alone (A and B), antiangiogenic agent (C and D), and anti–epidermal growth factor receptor agent (E and F) groups. The difference in nadir is the difference between the model-estimated mean relative tumor-size change at nadir (relative to baseline) in each contrast. The line indicates weighted regression; the sizes of the circles are proportional to the total sample sizes of the corresponding contrasts.

The associations between the differences in time to nadir and logHRs for OS, as well as between the differences in depth of nadir and logHRs for OS, are presented in Figure 2, with a weighted regression line. The estimated value of *R*^2^ was 0.63 (95% CI, 0.30-0.96) for the association between the treatment effects on time to nadir and OS, and 0.08 (95% CI, 0-0.37) for the association between the treatment effects on depth of nadir and OS. eFigure 3A in the Supplement presents the estimated values of *R*(*t*) that quantify the association at the individual level between RTSC and OS at time *t*. At all considered time points, *R*(*t*) values were 0.9 or larger. Thus, the plot indicates that RTSC values provide much information on a patient's OS.

### Anti-ANG Agents

For anti-ANG agent contrasts, data on 5390 patients enrolled in 6 trials were available for analysis. After excluding patients with no tumor-size information or with tumor-size measurements available only more than 24 months after randomization, 4854 (90.1%) of the patients could be analyzed (Table 1). Eleven contrasts could be formed, with median follow-up in each trial ranging from 14 to 31 months. eFigure 1B in the Supplement shows the OS curves for each of these contrasts, and the corresponding HRs are presented in Table 2. eFigure 2B in the Supplement presents the longitudinal RTSC profiles for these contrasts, and the corresponding estimates of treatment effects on time to nadir and on depth of nadir are presented in Table 2. All effects on time to nadir were positive, suggesting that the nadir for the experimental treatments took place later than for the control treatments. At the same time, all but 2 (for HORIZON III A and N016966 C) effects on depth of nadir were negative, suggesting that the experimental treatments led to a larger relative reduction in tumor size than the control treatments. This finding reflects that the RTSC profiles for the control arms exhibited a higher curvature than the profiles for the experimental arms (eFigure 2B in the Supplement).

The associations between treatment effects on time to nadir and depth of nadir and on OS are shown in Figure 2B. The estimated value of *R*^2^ was 0.25 (95% CI, 0-0.72) for the association between the treatment effects on time to nadir and OS and 0.06 (95% CI, 0-0.35) for the association between the treatment effects on depth of nadir and OS. eFigure 3B in the Supplement depicts the association at the individual level between RTSC and OS at time *t*. Values of *R*(*t*) become larger than 0.9 for *t* of approximately 6 months. Thus, the plot suggests that, initially, RTSC values provided relatively little information on a patient's OS. However, as additional information on tumor size was gathered over time during the first year of treatment, RTSC achieved a better predictive strength for OS, with no further gain in the subsequent year.

### Anti-EGFR Agents

Of 3081 eligible patients enrolled in 6 trials involving anti-EGFR agents, 2684 patients (87.1%) could be analyzed after excluding those without any tumor-size information or with tumor-size measurements available only more than 24 months after randomization (Table 1). These patients were grouped into 8 contrasts, and the median follow-up in each trial ranged from 10 to 47 months. eFigure 1C in the Supplement presents the OS curves for these contrasts. The corresponding HRs are reported in Table 2. eFigure 2C in the Supplement presents the longitudinal RTSC profiles for these contrasts, and the corresponding estimates of the treatment effects on time to nadir and depth of nadir are given in Table 2. Although the effects on time to nadir show some heterogeneity (range from −2.85 for COIN B to 2.40 for OPUS, excluding PACCE [C249] B), once again, all but 2 (for PACCE [C249] A and B) of the effects on depth of nadir were negative, suggesting that the experimental treatments led to larger tumor shrinkage than the control treatments. This finding reflects that the RTSC profiles for the experimental arms seem to be shifted down as compared with the control-arm profiles, while exhibiting roughly a similar curvature (eFigure 2C in the Supplement). An exception was the PACCE B comparison, for which the estimated RTSC profile for the control arm decreased, unlike for the experimental arm. As a consequence, the estimated time to nadir for the control arm was long (equal to 171.1 months) and resulted in treatment effects on time to nadir (−163.1) and depth of nadir (0.41) that were markedly different from the other comparisons (Table 2).

The associations between treatment effects are depicted in Figure 2E and F. All comparisons were taken into account, and the estimated value of *R*^2^ was 0.24 (95% CI, 0-0.83) for the association between the treatment effects on time to nadir and OS and 0.21 (95% CI, 0-0.78) for the association between the treatment effects on depth of nadir and OS. When the PACCE B comparison was excluded from the analysis, the estimates of *R*^2^ were 0.36 (95% CI, 0-0.97) for depth of nadir and 0.18 (95% CI, 0-0.74) for OS. eFigure 3C in the Supplement depicts the individual-level association between RTSC and OS at time *t*. At all considered time points, values of *R*(*t*) are smaller than 0.4, suggesting that RTSC provided little information on a patient's OS.

## Discussion

Given the continuum of care in mCRC, it becomes increasingly difficult to demonstrate gains in OS in first-line treatment trials. This difficulty has heightened interest in alternative strategies, such as adaptive designs^37^ and the use of surrogate end points, including those based on tumor measurements. The latter approach is contrary to the key finding from the present study that neither time to nadir nor depth of nadir can be considered a valid surrogate for OS using contemporary regimens for first-line therapy of mCRC. At best, time to nadir appears to display a moderate association with OS at the trial level with chemotherapy alone or combined with an anti-ANG agent, while depth of nadir appears to display a weak association with OS in all treatment classes. Another finding from this study is the apparent difference between the response kinetics of regimens that include an anti-ANG agent and those that involve an anti-EGFR agent.

The difference in tumor-growth kinetics between anti-ANG and anti-EGFR agents may warrant further exploration. Data presented in Table 2 and eFigure 2 in the Supplement suggest that the addition of an anti-ANG agent to chemotherapy is associated with a later, although not often deeper, nadir. Conversely, the addition of an anti-EGFR agent often produces a deeper nadir, with less-conclusive results about its timing of occurrence. These exploratory observations are based on a relatively small number of contrasts, but they may support the clinical impression that the addition of an anti-EGFR agent produces a larger influence on the depth of responses than the addition of an anti-ANG agent. Albeit subject to bias owing to the above-mentioned reasons, the often-divergent slopes after nadir between control and experimental arms as shown in eFigure 2 in the Supplement suggest that the tumor-growth kinetics with both classes of agents are not marked by a rebound effect after progression. The differences in tumor-growth kinetics among different classes of agents are also reflected on the individual-level associations between the RTSC and OS processes. For chemotherapy, it seems that RTSC may provide a strong prediction of a patient’s survival. For anti-ANG agents, a strong correlation might be inferred after the initial half-year of treatment. However, for anti-EGFR agents, the correlation appeared to be weak. These individual-level estimates depend largely on the form of the models applied and should be interpreted with caution.

### Strengths and Limitations

Strengths of this study are the large sample size and representativeness in terms of contemporary first-line therapy. Moreover, results of this study suggest that the dimensions of measurable tumor lesions can be modeled to provide information on tumor-growth kinetics. In this sense, our approach differs from the one used by Mansmann et al,^10^ who did not model tumor size as a function of time and did not estimate trial-level associations, which is a current requirement for surrogacy validation.^38^

This study has limitations. The chief limitation of this study is the absence of tumor measurements for all patients, which is a potential source of bias through exclusion of individuals with features that may differ systematically from those of included patients. Likewise, extended RAS testing was not available at the time that these trials were conducted, leading to a predictably small percentage of patients being falsely considered as having wild-type tumors. Moreover, no data were available on tumor sidedness or other potential prognostic or predictive molecular markers, such as the status of microsatellite instability, *BRAF*, or *HER2*. Limitations also apply to the model building, which is affected by the absence of postprogression measurements. Moreover, if progression is due to new lesions before the sum of target lesions has reached the nadir, there is increased uncertainty in the estimation of time to nadir and depth of nadir. Also, new lesions could not be included in the definition of RTSC, because the size of such lesions was not reported. In addition, the strength of the association between treatment effects on time to nadir or depth of nadir and on OS was assessed by using a linear regression model weighted by the sample size to account for the uncertainty in the estimated treatment effects. A methodologically more appropriate approach would be to take into account estimates of the SEs and correlation of the estimated treatment effects.^39^ However, obtaining such estimates for the joint model used in our analysis was not possible, because the model was fitted by using the expectation-maximization algorithm.

## Conclusions

Neither time to nadir nor depth of nadir appears to be an acceptable surrogate for OS. These findings are not surprising, given the weak trial-level association between conventional response rates and OS in mCRC, despite their association with OS at the patient level, both in mCRC and advanced breast cancer.^40,41^ This distinction indicates that achieving response may convey prognostic information for patients in clinical practice, but at the same time suggests that response-based end points cannot replace OS in clinical trials. In none of the treatment classes analyzed was the association between treatment effects strong enough to warrant reasonable precision of the prediction of the treatment effect on OS from the effect on time to nadir or depth of nadir. Such a reasonable precision of the prediction is currently considered the key requirement for a surrogate end point.^38^ Nevertheless, at least for chemotherapy and targeted agents, the use of response-based end points in early-phase trials has been helpful in selecting regimens for further testing in phase 3 trials. Moreover, in clinical practice, a deeper response may help in controlling symptoms and increase the chance of performing secondary resections. Therefore, the implications of these results for early drug development and clinical practice are unclear and warrant further studies. In addition, the findings of this study reinforce the need to develop more reliable end points that reflect tumor biology and patient benefit.

## Notes

Supplement. **eFigure 1.** Kaplan-Meier Overall Survival Curves for Each Contrast **eFigure 2.** Model-Based Estimated Longitudinal Profiles for Each Contrast **eFigure 3.** Individual-Level R_ind_ (Referred to as *R(t)* in the manuscript)
