# Supplementary material for: Evaluation of Continuous Tumor-Size–Based End Points as Surrogates for Overall Survival in Randomized Clinical Trials in Metastatic Colorectal Cancer
Source: JAMA Netw Open. 2019 Sep 20;2(9):e1911750. doi: 10.1001/jamanetworkopen.2019.11750 (PMC6755539; doi:10.1001/jamanetworkopen.2019.11750)
Supplement: Supplement. — eFigure 1. Kaplan-Meier Overall Survival Curves for Each Contrast eFigure 2. Model-Based Estimated Longitudinal Profiles for Each Contrast eFigure 3. Individual-Level Rind (Referred to as R(t) in the manuscript) [file jamanetwopen-2-e1911750-s001.pdf]

Supplementary Online Content

Burzykowski T, Coart E, Saad ED, et al; Aide et Recherche en Cancerologie Digestive Group. Evaluation of continuous tumor-size–based end points as surrogates for overall survival in randomized clinical trials in metastatic colorectal cancer. *JAMA Netw Open*. 2019;2(9):e1911750. doi:10.1001/jamanetworkopen.2019.11750

eFigure 1. Kaplan-Meier Overall Survival Curves for Each Contrast

eFigure 2. Model-Based Estimated Longitudinal Profiles for Each Contrast

eFigure 3. Individual-Level R_{ind} (Referred to as $R(t)$ in the manuscript)

This supplementary material has been provided by the authors to give readers additional information about their work.

© 2019 Burzykowski T et al. *JAMA Network Open.*

eFigure 1. Kaplan-Meier overall survival curves for each contrast.
Panel A. Chemotherapy alone.

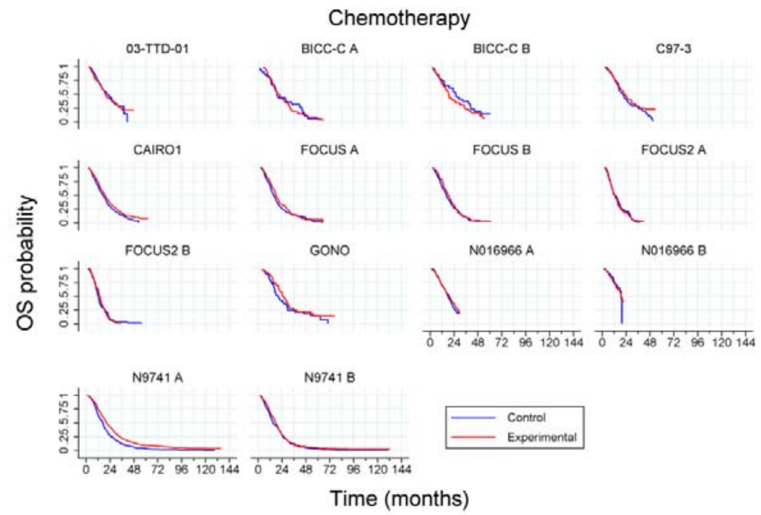

Panel B. Anti-angiogenic agents.

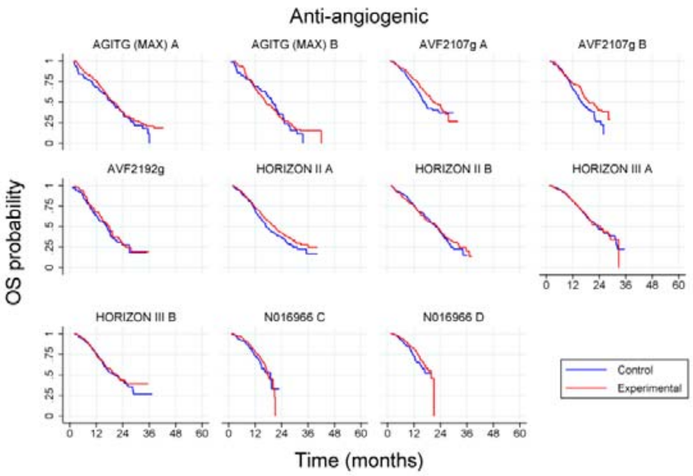

Panel C. Anti-EGFR agents.

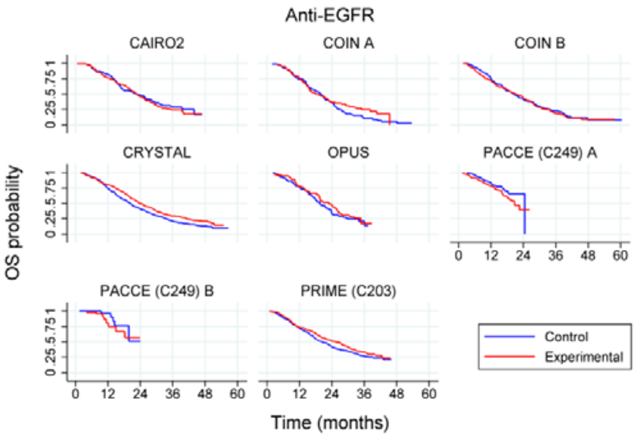

© 2019 Burzykowski T et al. *JAMA Network Open.*

eFigure 2. Model-based estimated longitudinal profiles for each contrast.
Panel A. Chemotherapy alone.

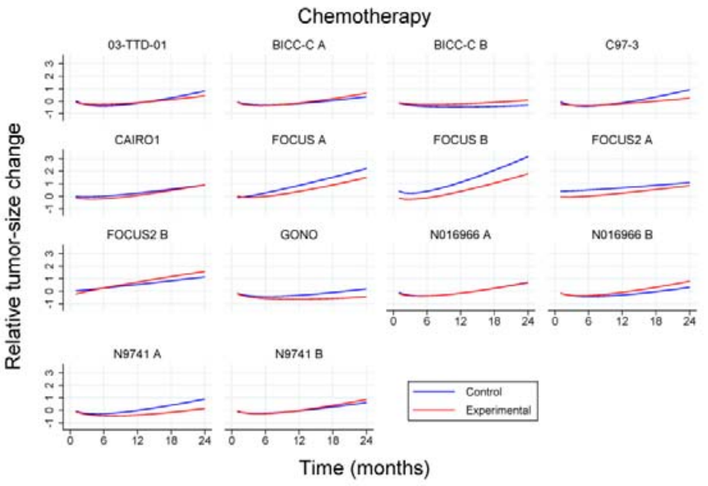

Panel B. Anti-angiogenic agents.

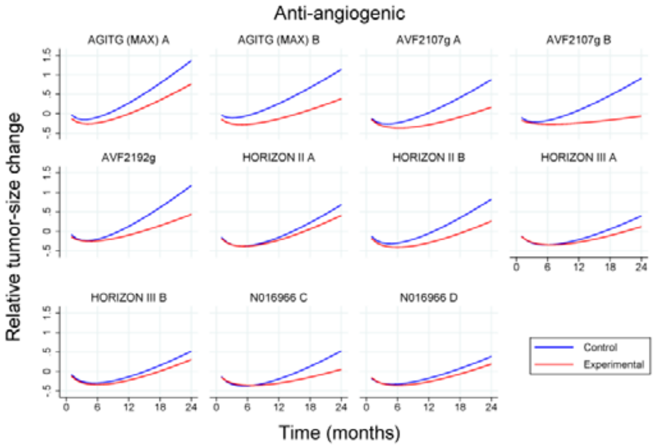

Panel C. Anti-EGFR agents.

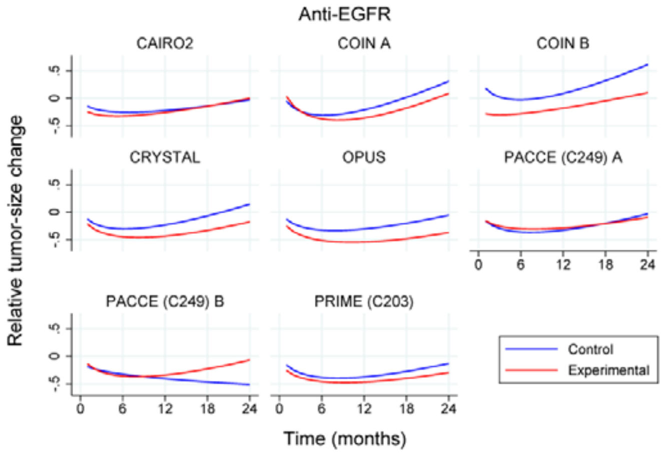

© 2019 Burzykowski T et al. *JAMA Network Open.*

eFigure 3. Individual-level R_{ind} (Referred to as *R*(*t*) in the manuscript).

Panel A. Chemotherapy alone.

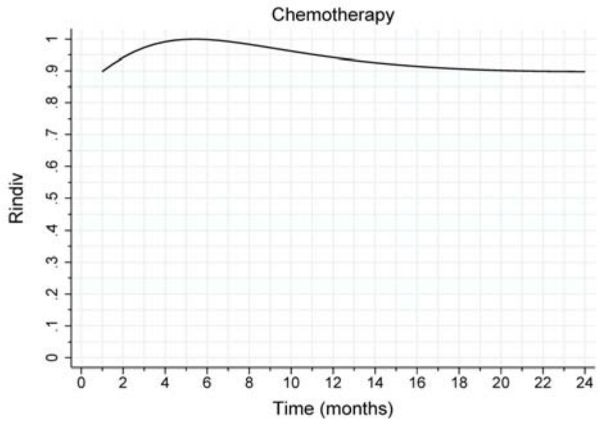

Panel B. Anti-angiogenic agents.

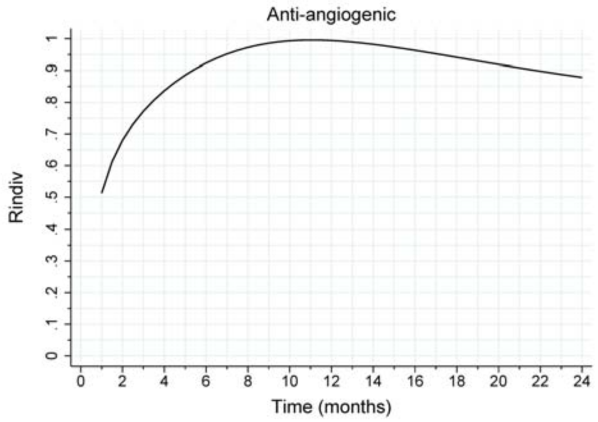

Panel C. Anti-EGFR agents.

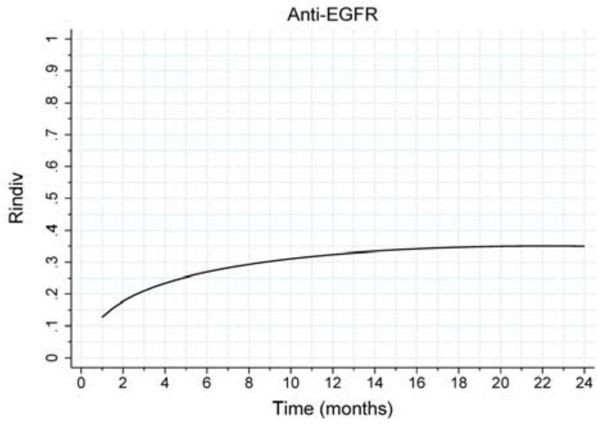

© 2019 Burzykowski T et al. *JAMA Network Open.*
